# Comparison of hearing performance in patients with borderline indication for active middle ear or cochlear implants: clinical outcomes to guide preoperative counseling and decision making

**DOI:** 10.1007/s00405-024-08491-6

**Published:** 2024-02-07

**Authors:** Constanze Herr, Timo Stöver, Uwe Baumann, Tobias Weissgerber

**Affiliations:** 1 Department of Otorhinolaryngology, Goethe University Frankfurt, University Hospital Frankfurt, Theodor-Stern-Kai 7, 60590 Frankfurt (Main), Germany; 2 Department of Otorhinolaryngology, Audiological Acoustics, Goethe University Frankfurt, University Hospital Frankfurt, Frankfurt (Main), Germany

**Keywords:** Cochlear implant, Vibrant soundbridge, Borderline indication, Speech perception, Counselling

## Abstract

**Purpose:**

The aim of the presented study was to compare the audiological benefit achieved in cochlear implant (CI) patients who, in principle, could still have been treated with an active middle ear implant (AMEI) with a group of AMEI users.

**Methods:**

Results of 20 CI patients with a pure-tone average (PTA) of 70 dB HL prior to surgery were compared with a group of 12 subjects treated with a Vibrant Soundbridge (VSB). Pre-surgical comparison included PTA for air conduction and bone conduction, maximum speech recognition score for monosyllabic words (WRSmax), and aided monosyllabic word recognition at 65 dB SPL. One year after surgery, aided monosyllabic speech recognition score at 65 dB SPL was compared.

**Results:**

Mean PTA for air conduction in the VSB group was significantly lower than in the CI group (4.8 dB, *Z* =  − 2.011, *p* < 0.05). Mean PTA for bone conduction in the VSB group was also significantly lower than in the CI group (23.4 dB, *Z* =  − 4.673, *p* < 0.001). WRSmax in the VSB group was significantly better than in the CI group (40.7%, *Z* =  − 3.705, *p* < 0.001). One year after treatment, there was no significant difference in aided speech perception in quiet between both subject groups.

**Conclusion:**

Comparison of the two methods showed equivalent results for both treatments in subjects with a borderline indication. Not only pure-tone audiometry results but, particularly, speech perception scores pre-surgery should be taken into account in preoperative counseling.

## Introduction

The treatment of patients suffering from sensorineural hearing loss offers a wide range of technical options. Depending on the degree of hearing loss, these consist of conventional hearing aids [1], active middle ear implants [2] or cochlear implants (CI) with either electric or electric-acoustic stimulation [3, 4]. The boundaries with regard to the respective indications of these methods are increasingly overlapping. Thus, the decision making process can lack certainty. Therefore, a particular challenge in clinical practice is the counseling of patients with a moderate to severe hearing loss, as in these cases it may still be useful to fit a hearing aid, or to “already” provide an active middle ear implant or even a CI.

In particular, the decision between a hearing device with acoustic amplification (i.e., hearing aid or middle ear implant) and a CI can be challenging. Using acoustically amplified hearing devices can be advantageous as it potentially offers a more natural sound perception. In addition, habituation usually is quick, as no special hearing rehabilitation is required. However, a progression of hearing loss may result in insufficient amplification with inadequate speech perception. In contrast, CI fitting involves increased surgical and technical effort, and requires intensive hearing rehabilitation [5]. On the other hand, CI fitting usually provides higher long-term stability of hearing.

Usually, the success of surgical treatment and, thus, the indication can only be reliably assessed retrospectively. Therefore, the aim of the presented study was to examine the audiological benefit achieved in patients who received CI treatment but who, in principle, could still have been treated with an active middle ear implant (mean sensorineural hearing loss of 70 dB HL or less). In our retrospective study, these patients were then compared to patients who received a Vibrant SoundBridge system (VSB, MED-EL, Innsbruck, Austria, [6]). As the floating mass transducer (FMT) of the VSB device can be coupled to various structures of the ossicular chain and the round window [7], the indication for the VSB was either conductive, mixed [8] or sensorineural hearing loss (especially in cases, were no hearing aid could be worn) [9].

The audiological results of both groups (VSB and CI) were compared in order to help future counseling of patients with similar "borderline indication" to select the ideal device system.

## Material and methods

The inclusion criteria of the CI group were individuals with a maximum mean sensorineural hearing loss of 70 dB HL (four-frequency pure-tone average PTA for air conduction, frequencies: 0.5/1/2/4 kHz) without any substantial conductive hearing loss (i.e. ≤ 15 dB) at the time before CI surgery. Twenty patients (mean age at surgery: 60.2 ± 16.5 years) from our database met this criterion. Demographic data of the CI group are shown in Table 1.

**Table 1:** Demographic data of the CI group with mean (pure-tone average, PTA) air conduction hearing loss (PTA_AC) and degree of conductive hearing loss

ID	Age at surgery [yrs]	Implant type	PTA_AC [dB HL]	PTA_AC-PTA_BC (conductive hearing loss) [dB HL]
1	70.1	Cochlear CI422	61.3	3.8
2	79.2	Cochlear CI422	63.8	3.8
3	61.8	Cochlear CI422	67.5	7.5
4	27.8	Cochlear CI522	65	8.7
5	37.8	AB HiRes90K MS	67.5	6.2
6	40.8	Cochlear CI532	68.8	10.0
7	46.0	Cochlear CI522	58.8	10.0
8	79.9	Cochlear CI522	62.5	2.5
9	78.2	Cochlear CI512	63.8	10
10	77.9	Cochlear CI512	67.5	12.5
11	67.7	Cochlear CI532	66.3	1.3
12	54.2	MED-EL Synchrony Flex28	70	6.2
13	56.3	AB HiRes Ultra 3D SlimJ	66.3	8.8
14	82.6	MED-EL Synchrony2 Flex28	70	10.0
15	51.8	Cochlear CI632	67.5	3.7
16	60.9	MED-EL Synchrony 2 Flex26	65	1.2
17	51.3	Cochlear CI632	67.5	3.7
18	56.3	MED-EL Synchrony 2 Flex26	63.8	10.0
19	81.3	AB HiRes Ultra 3D SlimJ	68.8	5
20	42.7	Cochlear CI622	62.6	5.1

The comparison group consisted of patients that received an active middle ear implant (VSB). The VSB group included 12 patients (mean age at surgery: 56.6 ± 16.4 years). These were fitted with either coupling of the VSB to the ossicular chain (n = 5) or round window (n = 7). Demographic data of the VSB group are shown in Table 2. There was no statistically significant difference in age between the two groups.

**Table 2:** Demographic data of the VSB group for different coupling sites with mean (pure-tone average, PTA) air conduction hearing loss (PTA_AC) and bone conduction hearing loss (PTA_BC)

ID	Age at surgery [yrs]	Implant type and coupling	PTA_AC [dB HL]	PTA_BC [dB HL]
1	68.5	VORP 502, RW	107.5	38.8
2	41.4	VORP 502, Incus	51.3	46.3
3	69.5	VORP 502, Incus	58.8	38.8
4	61.2	VORP 502, Incus	50	38.8
5	40.1	VORP 503, RW	57.5	28.8
6	59.0	VORP 503, Clip-Coupler	48.8	30
7	16.0	VORP 503, RW	37.5	11.3
8	54.3	VORP 503, SP	33.8	26.3
9	70.9	VORP 503, RW	65	48.8
10	63.8	VORP 503, RW	76.3	33.8
11	67.8	VORP 503, RW	98.8	45
12	66.8	VORP 503, SP	45	40

RW, round window; Incus, incus long process; SP, incus short process

For both groups (CI and VSB) the PTA for air conduction and bone conduction were compared before CI and VSB surgery, respectively. Furthermore, the maximum speech recognition score for monosyllabic words WRSmax (ipsilateral measurement, air conduction) and the aided speech recognition score for monosyllabic words (ipsilateral hearing aid, free-field measurement at 65 dB SPL) before CI or VSB fitting was assessed with the Freiburg monosyllable test [10]. One year after surgery, aided monosyllabic speech recognition score (either ipsilateral CI or VSB, free-field measurement at 65 dB SPL) was compared.

In the fitting session one year after surgery, subjects were asked to rate their subjective satisfaction in using their hearing device on a category scale between 1 (very unsatisfied) and 6 (very satisfied).

Ethical approval was waived by the local institutional review board (No. 288/17) in view of the retrospective nature of the study and all the procedures being performed were part of the routine care.

### Statistics

Nonparametric tests were utilized for statistical analyses of all PTAs and speech perception scores. The Wilcoxon test was applied for pairwise comparisons and the Mann–Whitney-U test was used for group comparisons. A p-value < 0.05 was considered as significant. IBM SPSS Statistics 27 (IBM, Armonk, New York) was used for the analysis.

## Results

**Fig. 1:**
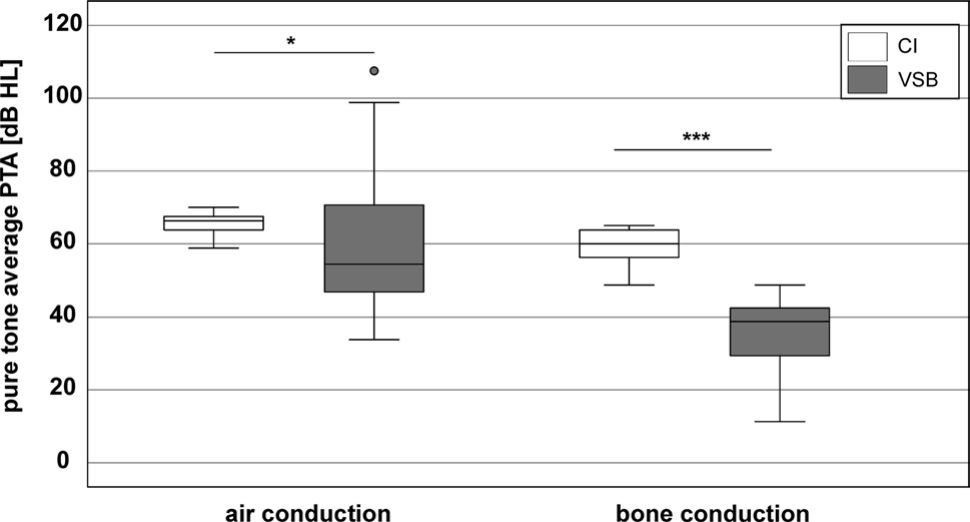
Pure tone average (PTA) pre surgery of the CI group (white) and VSB group (grey) for air conduction (PTA_AC) and bone conduction (PTA_BC). *: *p* < 0.05; ***: *p* < 0.001

Boxplots of PTA measurements pre-surgery for both subject groups were shown in Fig. 1. Mean PTA for air conduction in the CI group was 65.7 ± 3.0 dB HL, mean PTA in the VSB group was 60.9 ± 22.9 dB HL. PTA for air conduction in the VSB group was significantly lower than in the CI group (4.8 dB, *Z* =  − 2.011, *p* < 0.05).

Mean PTA for bone conduction in the CI group was 59.0 ± 4.4 dB HL, mean PTA in the VSB group was 35.6 ± 10.4 dB HL. PTA for bone conduction in the VSB group was significantly lower than in the CI group (23.4 dB, *Z* =  − 4.673, *p* < 0.001). This difference in PTA for bone conduction between subject groups was not surprising, since all types of hearing loss were included in the VSB group, whereas the typical indication for CI surgery is a sensorineural hearing loss.

**Fig. 2:**
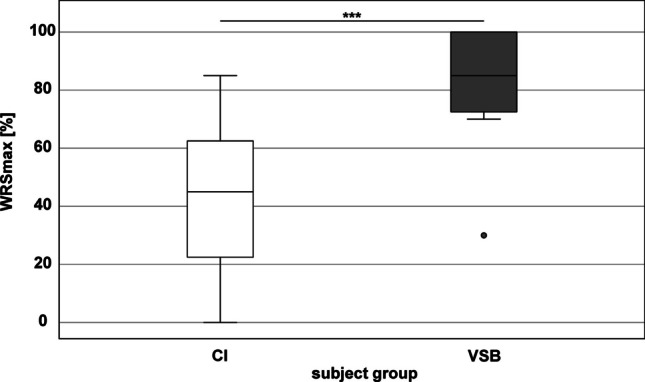
Preoperative percentage of maximum monosyllabic word recognition score (PBmax) pre surgery for the CI group (white) and VSB group (grey). There was a significant difference between subject groups in WRSmax (*p* < 0.001). ***: *p* < 0.001

**Fig. 3:**
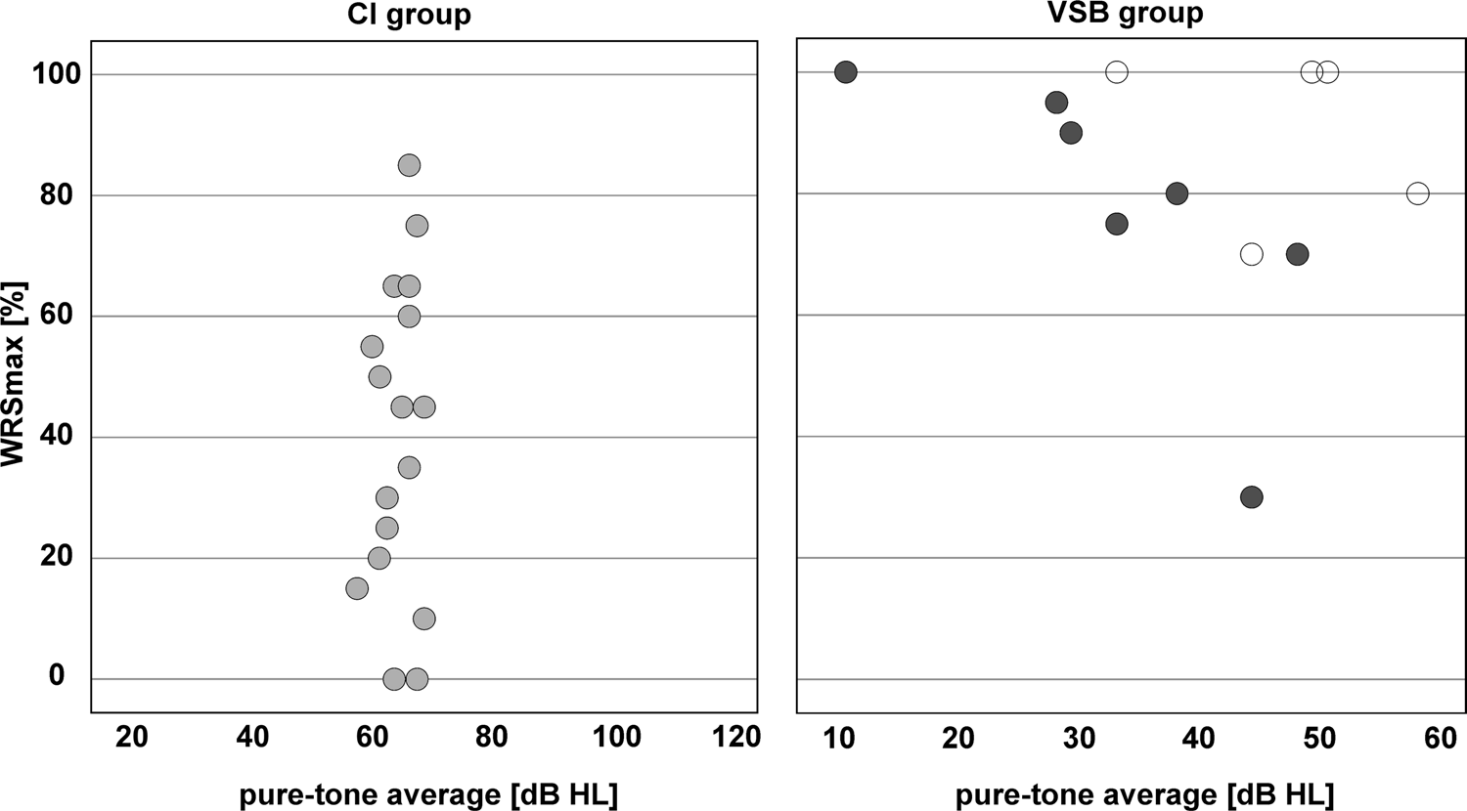
Scatter plots comparing the individual pure-tone average with WRSmax for the CI group (left) and VSB group (right) for round window coupling (dark grey circles) and ossicular chain coupling (white circles)

Comparison of maximum monosyllabic word recognition score (WRSmax) between the two subject groups prior to surgery is shown in Fig. 2. Mean WRSmax in the CI group was 41.8 ± 26.1%, mean WRSmax in the VSB group was 82.5 ± 20.4%. WRSmax in the VSB group was significantly better than in the CI group (40.7%, *Z* =  − 3.705, *p* < 0.001). Figure 3 shows scatter plots of the individual pure-tone average (air conduction) vs. WRSmax for the CI group (left) and VSB group (right).

**Fig. 4:**
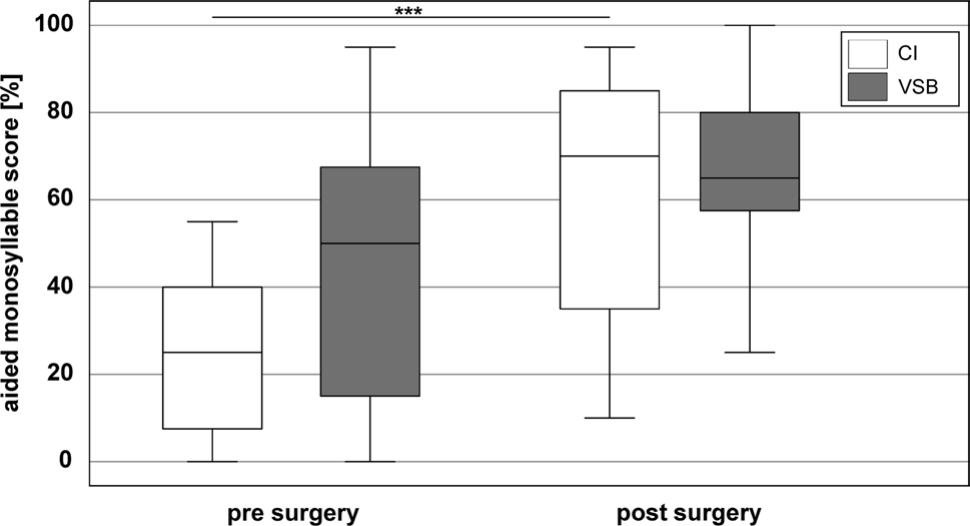
Aided monosyllabic score (measured at 65 dB SPL) pre-surgery with hearing aid and post-surgery for the CI group (white) and VSB group (grey). ***: *p* < 0.001

Boxplots of aided monosyllabic scores (measured at 65 dB SPL) pre-surgery with hearing aid and post-surgery with CI or VSB are shown in Fig. 4. Mean aided monosyllabic score with hearing aid was 25.4 ± 18.9% (CI group) and 42.9 ± 31.6% (VSB group). Possibly caused by the large variations within subject groups, no significant differences in speech perception with hearing aid pre-surgery were found between subject groups (*Z* =  − 1.762, *p* = 0.083).

Mean aided monosyllabic score one year after surgery in the CI group significantly increased to 61.4 ± 25.7% (*Z* =  − 3.746, *p* < 0.001). The improvement in the VSB group to 65.4 ± 23.1% failed to reach statistical significance (*Z* =  − 1.887, *p* = 0.059).

There was no significant difference in aided speech perception in quiet between both subject groups (*Z* = 0.039, *p* = 1.0). Within the VSB group there was no significant difference in speech perception between coupling methods (round window: 65 ± 29.6%, incus: 66 ± 12.5%; *Z* =  − 0.498, *p* = 0.64).

There was no correlation between pre-operative pure-tone average and post-operative monosyllabic word recognition scores with CI for both subject groups.

**Fig. 5:**
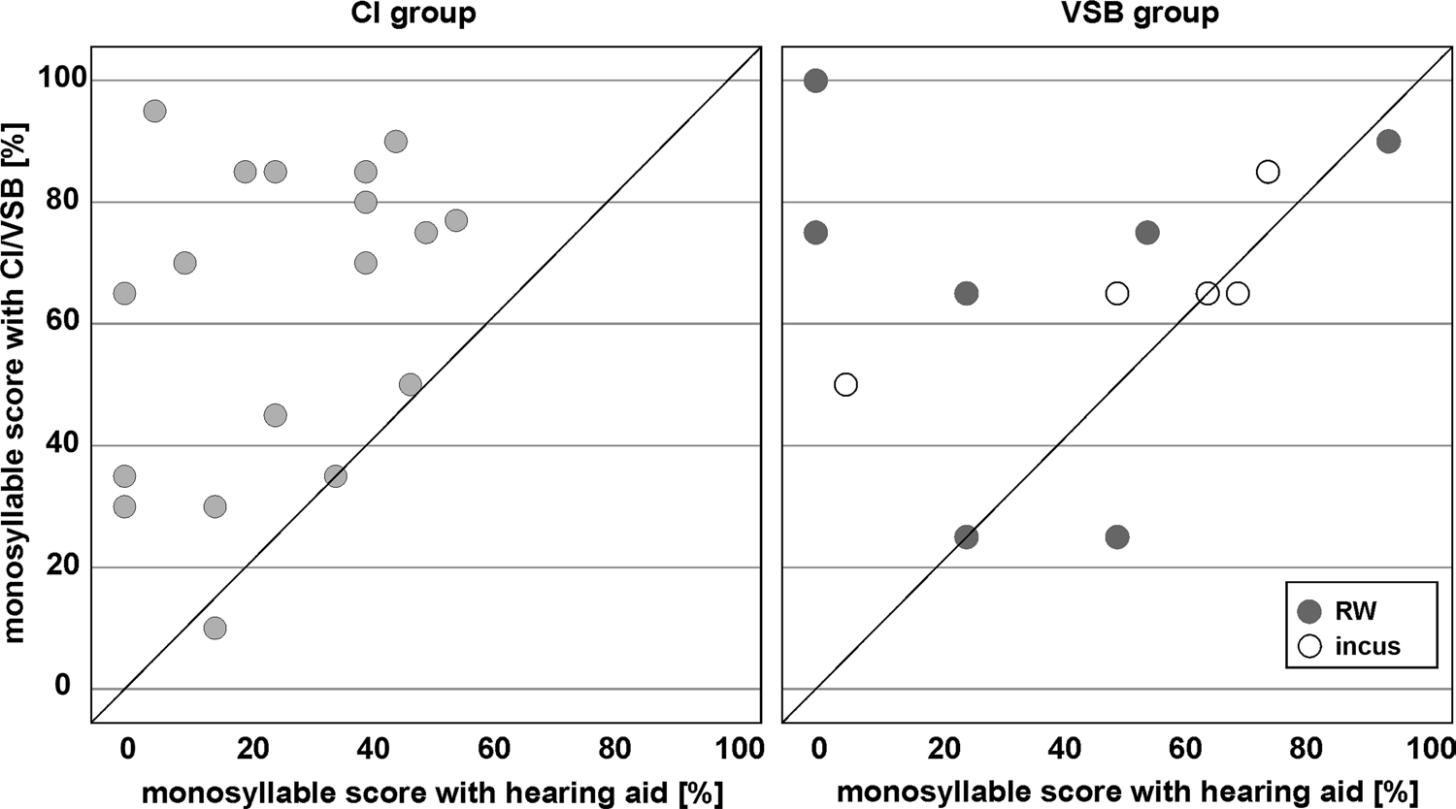
Scatter plots comparing the individual aided speech perception scores (measured at 65 dB SPL) pre-surgery with hearing aid and post-surgery for the CI group (left) and VSB group (right) for round window coupling (dark grey circles) and ossicular chain coupling (white circles)

Figure 5 shows scatter plots comparing the individual aided speech perception scores pre-surgery with hearing aid scores and 1 year post-surgery for the CI group (left) and VSB group (right). Three out of the 20 CI users had monosyllables scores which were only in the range of their performance with hearing aid pre-surgery. All other subjects showed improved speech perception by using the CI. No subject had speech perception scores with CI which were worse than with the hearing aid pre-surgery.

4 out of the 12 VSB users (two with round window coupling and two with ossicular chain coupling) had monosyllables scores which were only in the range of their performance with hearing aid pre-surgery. One VSB user with round window coupling showed even worse speech perception scores with VSB compared to the hearing aid score pre-surgery.

Data about patient’s satisfaction rating was available in 17/20 CI subjects and 9/12 VSB subjects. Mean satisfaction score was 4.8 ± 1.0 (CI group) and 4.7 ± 1.6 (VSB group) showing a general satisfaction with the hearing device in both subject groups. There was no significant difference between subject groups in their subjective satisfaction in hearing with their device (Z =  − 0.2; *p* = 0.87).

## Discussion

The care of patients with moderate to severe hearing loss presents a particular challenge in clinical practice with regard to the selection of the optimal method of care. This is due to the overlapping indication areas for conventional hearing aids, implantable hearing aids and cochlear implants.

Currently, the Vibrant Soundbridge implant is the most frequently used active middle ear implant system introduced almost 3 decades ago [6]. The application of this system is sensorineural hearing loss (typically with incus coupling or with stapes coupler) as well as conductive and mixed hearing loss (typically with round window coupling). According to the manufacturer, the maximum inner ear hearing loss should not exceed 45–65 dB HL (frequency-dependent) for mixed and conductive hearing loss and 65–85 dB HL (frequency-dependent) for pure sensorineural hearing loss [11].

In contrast to this, the indication of a cochlear implant has to be considered. While originally a complete loss of hearing and speech perception was a prerequisite, the introduction of the recent Cochlear Implant Clinical Practice Guideline published in 2021 [3, 12] has led to a redefinition of the indication in Germany. In the meantime, hearing thresholds but, particularly, speech perception in quiet are the relevant reference values for the CI indication. According to this guideline, the indication is primarily based on the speech perception scores achieved preoperatively with hearing aids. Accordingly, the patient should be counseled about the option of CI fitting if the aided monosyllabic word recognition score (Freiburg monosyllables at 65 dB SPL, [10]) is worse than or equal 60%, even with optimized hearing aid fitting [3].

Counseling patients should not be based solely on indication guidelines provided by the device manufacturer, but should take into account the outcome observed in already treated patients. It was shown in retrospective studies that WRSmax is the most important predictor of CI outcome in subjects with pre-operative residual hearing [13, 14]. WRSmax was the factor which explained 27% of the differences obtained in speech perception outcomes in quiet with CI [13, 15]. But only a few subjects considered in the aforementioned studies were also in the indication range for a VSB. Therefore, the aim of this work was to compare the audiological outcomes in patients with a so-called “borderline indication” who received a CI but were also still potential (but also borderline) candidates for an active middle ear implant. These data were compared to the outcomes after VSB fitting of a comparison group. All patients included in the study were initially fitted with a conventional hearing aid and hearing aid fitting was optimized prior to the patients’ decision for either CI or VSB implantation.

A significant improvement in speech perception in quiet (at 65 dB SPL) was achieved in both groups as a result of the respective fitting. Examination of the speech test results achieved 12 months after first fitting showed comparable group results for VSB and CI treatment. This is particularly interesting because the CI group could in principle have been fitted with a VSB as well since the indication for a VSB given by the manufacturer is solely based on pure-tone audiogram without taking the maximum speech recognition score for monosyllabic words into account. Our results in subjects with a borderline indication show no statistical disadvantage in CI fitting compared to VSB fitting. Furthermore, subjective satisfaction with the hearing device was comparable in both subject groups.

In order to evaluate these results in a differentiated way, the preoperative audiological baseline conditions of the two groups have to be compared. The analysis of the pre-operatively assessed pure-tone audiometry showed significantly worse hearing thresholds in the CI group compared to the VSB group. Furthermore, maximum monosyllabic speech recognition score was significantly worse in the CI group. Nevertheless, the achieved speech test results 12 months post-surgery did not differ significantly between the two groups. One explanation could be that CI patients with a preoperative residual hearing, i.e., a borderline indication, can achieve very good results. A comparison of the mean results of the CI group studied here indeed showed better speech test results compared to average data from the literature [16]. This can be seen as a strong argument for an early CI treatment when residual hearing is still present and not only treatment in complete deafness.

Another explanation for the comparable results in VSB and CI could also be the non-optimal use of the active middle ear implant. It is possible that the VSB group does not achieve the maximum benefit from residual hearing because optimal coupling of the system to the ossicular chain or round window was not achieved. This could have anatomical as well as surgical reasons. Evidence for this interpretation can also be obtained from consideration of the preoperative audiological findings. There was a significant difference between the air and bone conduction hearing thresholds of 25.3 dB in the VSB group, whereas no significant differences were found in the CI group. Likewise, there was a significant difference in preoperative bone conduction hearing thresholds between VSB and CI group. This means that, on average, a conductive hearing loss was more frequent in the VSB group. However, this is not surprising, since the reason for fitting the VSB to the patients concerned was to achieve a signal transmission to the inner ear structures by means of individual coupling options of the floating mass transducer (e.g. to the round window membrane) in case of middle ear pathology and the resulting conductive hearing loss [17, 18].

One aspect of particular relevance in patient counseling is the long-term benefit of the hearing device and the potential progression of hearing loss. It was shown that the VSB is a technically reliable active middle ear implant with long-term stability and low complication rates [19]. The same holds for cochlear implant devices [20]. However, taking progression of hearing loss into account, VSB treatment in subjects with higher preoperative hearing loss (i.e. borderline-indication) and younger age at the time of VSB implantation might need a further CI surgery within the upcoming eight years due to progression [21]. Brkic and coworkers reported that during an observation interval of 18 years, 18.5% of all VSB devices were replaced by a CI due to a progression in hearing loss [21]. They also reported that the mean hearing loss in this subject cohort was already higher prior to VSB surgery than in the entire population of VSB subjects. This suggests that progression of hearing loss is of special importance in the counselling of candidates with a borderline indication.

None of the subjects of the VSB group in the presented study had a severe progression of hearing loss leading to a VSB explantation to date. In our whole population of VSB subjects implanted within the last 16 years (n = 55) the VSB device was explanted in two subjects (i.e. 4%) due to progression of hearing loss, who were then provided with a CI.

Whereas hearing performance with both CI and VSB would be potentially deteriorated with increasing age by neural degeneration and cognitive decline to the same extend, a CI fitting would be the more stable and reliable treatment in the long-term considering age-related progression of inner ear hearing loss. This aspect should also be considered when counseling older patients.

### Study limitations

A critical aspect of the presented work is that the assessment of hearing performance was purely retrospective, and thus only the data documented in the medical records could be used. Therefore, outcome assessment was limited to monosyllabic speech perception in quiet. According to the current Clinical Practice Guideline, this is currently the most important variable for determining the indication and also for assessing the success of a fitting with a hearing aids or an implantable hearing systems (e.g. [3] for CI). Nevertheless, it cannot be excluded that using other test conditions, e.g. adaptive speech tests in noise, different results could be obtained by a CI or VSB treatment. The same applies to the assessment of sound quality and music perception or even listening fatigue, which could also differ between the two groups. Answering these questions was not the subject of this study and thus reserved for future, prospective study designs. However, in the study presented here, the results based on monosyllabic speech perception in quite showed no significant difference between VSB and CI results.

Another potential weakness of our study could be to the non-optimal coupling of the VSB-FMT. Although the VSB patients showed significantly better pure-tone thresholds and maximum monosyllabic word recognition scores (WRSmax) compared to the CI group, the better residual inner ear function could not be used optimally in some subjects of the group of VSB patients. This could be due to technical or surgical reasons, since the coupling of the VSB-FMT is a central aspect for the optimal hearing performance with VSB. Even if this would be the case, what ultimately counts for patients is the postoperative outcome. Here, it has to be stated that in our study no significant difference could be demonstrated with regard to the achieved monosyllabic speech perception in a borderline indication after VSB or CI fitting.

## Conclusion

In the study presented here, we were able to show that patients with moderate to profound hearing loss achieved postoperatively significant improvement in speech perception in quiet with either VSB or CI fitting. Comparison of the two methods shows equivalent results for both treatments in a borderline indication where, from an audiological perspective, both methods could in principle be used. Against the background of the age-related progression of hearing loss, CI thus represents a comparably successful and long-term stable treatment in this patient group. The history of pure-tone audiometry results and, particularly, speech perception scores pre-surgery should be taken into account in the preoperative counselling in order to select the most suited device for hearing rehabilitation.

## Data Availability

Individual data of study measurement results are available by request to the corresponding author.
